# Prevalence of Undiagnosed Risk Factors in Patients with First-Ever Ischemic Stroke Treated at MUHC: A Retrospective Analysis

**DOI:** 10.3390/jpm15090433

**Published:** 2025-09-09

**Authors:** Shorog Althubait, Heather Perkins, Robert Cote, Theodore Wein, Jeffrey Minuk, Eric Erhensperger, Liam Durcan, Aimen Moussaddy, Lucy Vieira

**Affiliations:** 1 Neurology, College of Medicine, King Khalid University, Abha 61413, Saudi Arabia; 2 Department of Neurology and Neurosurgery, McGill University, Montreal, QC H3A 0G4, Canada; heather.perkins@mcgill.ca (H.P.); robert.cote@mcgill.ca (R.C.); theodore.wein@mcgill.ca (T.W.); eric.erhensperger@mcgill.ca (E.E.); liam.durcan@mcgill.ca (L.D.); aimen.moussaddy@mcgill.ca (A.M.); lucy.vieira@mcgill.ca (L.V.)

**Keywords:** ischemic stroke, undiagnosed risk factors, hypertension, diabetes mellitus, hyperlipidemia, atrial fibrillation, family doctor, primary care, geographic variation

## Abstract

**Background:** Ischemic stroke is a leading cause of morbidity and mortality worldwide. Despite established prevention strategies, many patients present with previously undiagnosed vascular risk factors (URFs) at the time of their first-ever ischemic stroke, suggesting missed opportunities for early detection. In Canada, particularly in Quebec, access to primary care is inconsistent, and a substantial proportion of the population lacks attachment to a family doctor (FD). **Objective:** This study aimed to determine the prevalence of URFs among patients with first-ever ischemic stroke and to evaluate the relationship between URFs, geographic region, and access to primary care in Quebec, Canada. We hypothesized that patients without an FD would have a higher prevalence of URFs. **Methods:** We conducted a retrospective chart review of 610 patients admitted with first-ever ischemic stroke to the McGill University Health Center (MUHC) between 2014 and 2017. Data collected included demographics; known and undiagnosed stroke risk factors such as hypertension (HTN), diabetes mellitus (DM), hyperlipidemia (HLD), and atrial fibrillation (AF); FD status; and geographic location based on postal code. **Results:** Among the 610 patients, 136 (22.3%) had at least one URF. The most common URF was HLD (14.3%), followed by HTN (6.2%), AF (1.6%), and DM (0.1%). Of 609 patients with available data, 146 (23.97%) lacked an FD. Patients without an FD were significantly more likely to have undiagnosed HTN (7.6% vs. 2.1%, *p* = 0.008). No significant differences were observed for the other URFs. Geographic variation was noted in both URF prevalence and FD access, but regional differences were not statistically significant. **Conclusions:** Our findings support the hypothesis that a lack of an FD is associated with a higher prevalence of undiagnosed HTN in ischemic stroke patients. Targeted screening and improved access to primary care, particularly in underserved regions, may help to reduce the burden of preventable stroke by facilitating the earlier identification and management of modifiable risk factors.

## 1. Introduction

Ischemic stroke remains a leading cause of morbidity and mortality globally, representing a significant burden on healthcare systems and impacting quality of life for millions of individuals [1]. The complexity of stroke pathophysiology and its multifaceted risk factors necessitate a comprehensive understanding to improve prevention and management strategies. Among the numerous risk factors associated with ischemic stroke, hypertension (HTN), diabetes mellitus (DM), hyperlipidemia (HLD), and atrial fibrillation (AF) are frequently cited [1]. These conditions not only elevate the risk of stroke but also contribute to its severity and outcome. Despite advances in stroke prevention and treatment, many individuals present with undiagnosed risk factors (URFs) at the time of their first ischemic stroke, highlighting gaps in primary prevention strategies [2].

Undiagnosed risk factors refer to conditions that were either previously unknown to the patient or not yet identified by healthcare providers prior to the stroke event. Identifying these risk factors during an inpatient evaluation for stroke provides a critical opportunity for secondary prevention and highlights deficiencies in primary healthcare and screening processes. This especially holds relevance for young adults, as research on patients with first-ever stroke noted that younger patients tended to be unaware of their HTN prior to stroke [3]. Further, medication adherence is usually lower in younger patients, even after they have been diagnosed with chronic conditions [4]. Around 10% of ischemic strokes occur in individuals under 45, with major long-term socioeconomic effects [5,6,7]. Moreover, stroke prevention leads to more quality-weighted life-year gain in younger patients than in the elderly. Studies have shown that the presence of URFs can significantly impact stroke outcomes, stressing the need for proactive screening and management strategies aimed at mitigating these risks before a stroke occurs.

Hypertension is widely recognized as the most significant modifiable risk factor for ischemic stroke, with numerous studies demonstrating its association with increased stroke risk [2]. Similarly, DM and HLD are well-established stroke risk factors that contribute to atherosclerotic disease, which can lead to ischemic events. Atrial fibrillation, a common arrhythmia, increases the risk of stroke due to the potential for embolic events. Despite the established associations between these risk factors and stroke, many individuals present with undiagnosed or inadequately managed conditions at the time of their first stroke [2]. This suggests that primary prevention efforts, including regular screening and management of risk factors, are not reaching all at-risk individuals effectively.

In addition to individual risk factors, access to primary healthcare plays a crucial role in stroke prevention [8]. Having a family doctor (FD) can significantly impact the management and early detection of risk factors. Individuals without an FD may face challenges in accessing timely and comprehensive care, potentially leading to undiagnosed or poorly managed risk factors. In Canada, particularly in Quebec, access to primary care is a well-documented challenge. Reports indicate that a significant number of Quebec residents lack regular access to an FD, which can hinder opportunities for preventive care and the early detection of health risks. The provincial healthcare system has struggled to recruit and retain primary care providers, especially in specific geographic areas. This shortage contributes to delays in routine health assessments and the management of chronic diseases [9]. Geographic disparities in healthcare access further exacerbate these issues, as individuals living in underserved areas may experience greater difficulties in obtaining regular health evaluations and preventive care.

Although previous research has examined undiagnosed vascular risk factors and disparities in stroke care, there has been limited exploration of how these factors intersect with access to primary care and geographic variations within a universal healthcare system. To our knowledge, this study is one of the first to investigate the relationship between undiagnosed risk factors, the status of having a family doctor, and regional disparities among patients experiencing their first ischemic stroke in Quebec.

This study aims to address these gaps by evaluating the prevalence of URFs among patients with first-ever ischemic stroke treated at McGill University Health Center (MUHC). McGill University Health Center serves a diverse patient population, providing a unique setting for investigating the prevalence of URFs among patients with first-ever ischemic stroke. In the Canadian healthcare system, access to specialized care typically requires a referral from a primary care provider. As a result, having an FD is crucial for the timely identification and management of health risk factors. Without an FD, individuals may miss important opportunities for prevention and early intervention. Given the varying access to healthcare services and the distribution of primary care resources, examining the prevalence of URFs at MUHC can offer insights into the broader public health implications of stroke prevention and management. This is particularly relevant in the context of healthcare access disparities, as individuals without an FD or residing in underserved areas may experience delays in diagnosis and treatment of risk factors. We seek to determine the frequency of URFs in this patient population and explore the association between having an FD and the presence of URFs. Additionally, we will investigate geographic variations in the prevalence of URFs and access to primary care services. By examining these factors, we hope to identify areas for improvement in stroke prevention and highlight the importance of integrating comprehensive risk factor management into routine clinical practice.

The findings of this study have the potential to inform public health strategies and clinical guidelines by emphasizing the need for enhanced screening and management of stroke risk factors. Furthermore, understanding the role of primary care access in stroke prevention can guide policy interventions aimed at improving healthcare access and reducing disparities in stroke care. Ultimately, this research contributes to the broader goal of reducing the incidence of ischemic stroke through targeted prevention and improved healthcare delivery.

## 2. Methodology

### 2.1. Study Design and Setting

This study was a retrospective cross-sectional study conducted at McGill University Health Centre (MUHC) in Canada, focusing on first-ever acute ischemic stroke patients. The study aimed to evaluate the prevalence of URFs among these patients and assess the association between URFs and having an FD, including potential disparities based on geographic location.

### 2.2. Participants

We included all adult patients aged 18 and above who experienced a first-ever ischemic stroke and were treated at MUHC between January 2014 and December 2017. A total of 610 patients met the inclusion criteria and were enrolled in the study. Patients who had a prior history of ischemic stroke or transient ischemic attack (TIA), were under the age of 18, had incomplete medical records, or missing key data (e.g., risk factor history or postal code), presented with stroke mimics (e.g., seizures, migraines), were not admitted to a MUHC facility during the study timeframe, or diagnosed with hemorrhagic stroke or other non-ischemic cerebrovascular conditions were excluded. Medical records were reviewed to identify relevant cases and assess the presence of both diagnosed and undiagnosed risk factors. A convenience sampling approach was employed, encompassing all eligible patients who met the inclusion criteria during the study period.

### 2.3. Data Collection

Data were collected retrospectively from the electronic medical records of MUHC. The inclusion period was from 1 January 2014, to 31 December 2017. This 4-year window was selected to allow for a robust sample size while reflecting a stable period in diagnostic and treatment protocols for acute ischemic stroke. Medical records were systematically screened to extract information on a range of risk factors associated with ischemic stroke. The risk factors assessed included: Hypertension (HTN), Diabetes Mellitus (DM), Hyperlipidemia (HLD), Atrial Fibrillation (AF), Smoking Status, Obstructive Sleep Apnea (OSA), Coronary Artery Disease (CAD), Peripheral Artery Disease (PAD), and Transient Ischemic Attack (TIA).

The determination of URFs was based on documentation by treating physicians indicating newly identified risk factors during the inpatient evaluation for stroke. These risk factors were truly undiagnosed as they were not reported by healthcare providers and not reported by patients.

To facilitate geographic analysis while maintaining confidentiality, patient postal codes were grouped into six Area Codes that correspond to real-world administrative health regions within the Montreal area. Specifically, Area Code 1 represents the De Ouest region (COMTL), Area Code 2 corresponds to Du Centre-Ouest (CCSMTL), Area Code 3 includes Du Centre-Sud (CCSMTL), Area Code 4 comprises Du Nord (CMNTL), Area Code 5 includes De Est (CEMTL), and Area Code 6 encompasses patients residing outside the designated Montreal health regions. This categorization was derived from the Integrated Health and Social Services Centres (CIUSSS) boundaries used in Quebec’s healthcare system. The use of Area Codes enabled standardized, anonymized comparisons across regions with varying access to primary care services and different prevalence levels of undiagnosed risk factors. This structured grouping also addressed inconsistencies in regional labeling and allowed for a clearer interpretation of geographic disparities in the analysis.

### 2.4. Geographic Categorization

**Figure 1:**
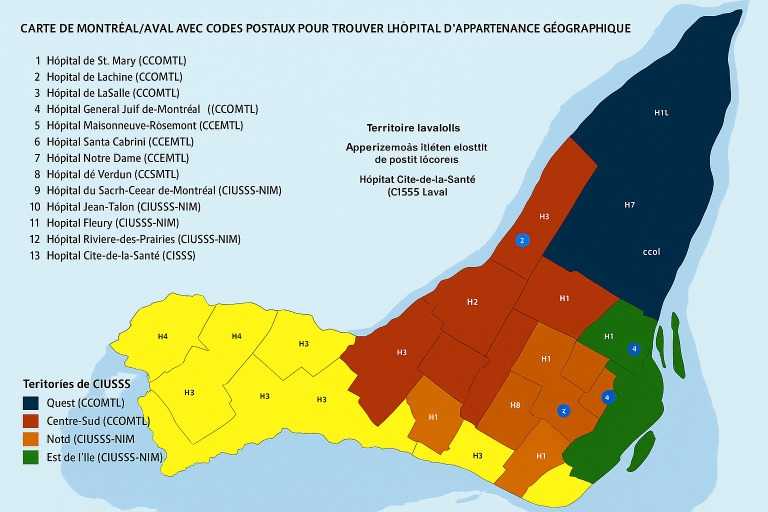
A Map of Montreal with Postal Codes to Locate the Geographically Assigned Hospital.

Postal codes from hospital registration records were mapped using forward sortation areas (FSAs), defined by the first three characters of Canadian postal codes (Figure 1). These FSAs were grouped into two broader geographic zones, Area 1 and Area 2, to protect patient confidentiality. Area classification was based on access to primary care services, socioeconomic indicators (like income quintiles, immigrant density, and education levels from census-linked FSA data), and insights from MUHC’s health planning team regarding underserved and better-served regions in the Greater Montreal Area. Area 1 included FSAs with good access to family physicians and higher socioeconomic status, while Area 2 encompassed underserved neighborhoods facing significant barriers to primary care and a higher prevalence of chronic disease risk factors. This categorization enabled an exploration of geographic disparities in undiagnosed risk factors and access to preventive care.

### 2.5. Statistical Analysis

Descriptive statistics were utilized to summarize the demographic characteristics and the distribution of risk factors within the sample. Cross-tabulation was employed to assess the relationship between having an FD and the presence of URFs across different postcode regions. Additionally, we compared the prevalence of URFs among individuals with and without an FD to identify any significant patterns or gaps in healthcare coverage. The chi-square test was used to assess the association between categorical variables, and the *p*-value was considered significant at 0.05. All statistical analyses were performed using IBM SPSS Statistics Version 26.0.

### 2.6. Ethical Considerations

The study was conducted in accordance with ethical standards, and patient confidentiality was maintained throughout the research process. The study protocol was approved by the institutional review board (IRB) of MUHC.

## 3. Results

A total of 610 patients presenting with first-ever ischemic stroke were enrolled in the study. The mean age of the cohort was 65 years, with a standard deviation of 15 years, indicating a wide age distribution among participants. The study population comprised 353 males (57.9%) and 257 females (42.1%), demonstrating a slightly higher prevalence of ischemic stroke in males compared to females (Table 1).

The most prevalent stroke risk factors at admission were HTN (361/610; 59.2%) and HLD (354/610; 58.0%), followed by DM (163/610; 26.7%). AF was present in 92 patients (15.1%), and smoking was reported in 102 patients (16.7%). Less common risk factors included OSA (27/610; 4.4%), CAD (91/610; 14.9%), PAD (6/610; 1.0%), and prior TIA (23/610; 3.8%) (Table 2).

Regarding the URFs, the most common URF was HLD (87/610; 14.3%), followed by HTN (38/610; 6.2%). Newly detected AF was observed in 10 patients (1.6%), while DM was newly identified in only one patient (0.1%) (Table 3).

Among the 609 patients with available data on primary care status, 463 individuals (76.0%) reported having a family doctor (FD), while 146 (24.0%) did not. This indicates that nearly one-quarter of the cohort lacked an FD, potentially contributing to delayed identification and management of vascular risk factors. Information was missing for only one patient (0.16%) (Table 4).

Patient distribution by health administrative postal regions revealed that the largest proportion resided in the De Ouest (COMTL) region (183/610; 30.0%), followed by Du Centre-Ouest (CCSMTL), with 125 patients (20.5%), and Du Centre-Sud (CCSMTL), with 90 patients (14.8%). Smaller proportions were from Du Nord (CMNTL) (26; 4.3%) and De Est (CEMTL) (37; 6.1%). Additionally, 149 patients (24.4%) resided outside the mapped postal regions (Table 5).

Across all postal regions, the majority of patients reported having a family doctor (FD), although regional differences were observed. In De Ouest, 81.42% of patients had an FD, representing the highest proportion, while De Est had the lowest at 67.57%. The highest proportions of patients without an FD were found in De Est (32.43%) and Du Centre-Ouest (29.03%), indicating potential regional disparities in access to primary care (Table 6).

The prevalence of URFs varied across health administrative regions. For HTN, rates ranged from 4.70% in patients residing outside the mapped regions to 8.80% in Du Centre-Ouest. Undiagnosed HLD was most prevalent in De Est (18.92%) and Du Centre-Ouest (16.80%). Newly identified AF was rare across all regions, with no cases observed in Du Nord or among patients from outside mapped areas. Undiagnosed DM was identified in only one region—Du Centre-Ouest (0.80%)—and was absent in all others. Although none of these regional differences reached statistical significance (all *p* > 0.05), the observed patterns may indicate disparities in early detection of vascular risk factors and highlight potential gaps in primary healthcare delivery by geographic area (Table 7).

The relationship between URFs and FD access revealed that patients without an FD were more likely to present with undiagnosed HTN (2.05% vs. 7.56%, *p* = 0.008). For other risk factors—DM, HLD, and AF—there were no statistically significant differences between those with and without an FD. Nevertheless, 30.90% of all patients with any URF lacked an FD, suggesting that reduced access to primary care may contribute to missed diagnoses, particularly for HTN (Table 8).

## 4. Discussion

This study aimed to identify the prevalence of URFs among patients presenting with their first-ever ischemic stroke and to explore the role of primary care access in managing these risk factors. Our findings indicate that over one-fifth of patients had at least one URF, predominantly HLD, HTN, and AF, which underscores ongoing deficiencies in routine risk screening and management in primary care settings. This discussion will contextualize these results within the existing literature, examine the implications for stroke prevention, and propose strategies to address the identified challenges in healthcare access and risk factor management.

### 4.1. Prevalence of Undiagnosed Risk Factors

Our study found that 22.13% of patients presenting with their first-ever ischemic stroke had at least one URF, predominantly HLD (14.3%), HTN (6.2%), and AF (1.6%). This aligns with a recent analysis conducted by Rêgo et al. [2] which reported that of 4354 first-ever ischemic strokes, about 26% of patients had undiagnosed vascular risk factors, 61.4% had dyslipidemia, 23.7% had hypertension, and 10.2% had atrial fibrillation, supporting the predominance of these conditions in previously unrecognized stroke risk.

The high prevalence of undiagnosed HLD is consistent with the American Heart Association findings, which report that a significant proportion of individuals with elevated cholesterol levels remain undiagnosed until they suffer from a cardiovascular event [10]. However, despite the high prevalence of HLD among the participants in our study, it was not statistically significant. Hyperlipidemia contributes to atherosclerosis and subsequent plaque formation, which can lead to vessel occlusion and stroke [11]. This underscores a persistent gap in lipid screening and early intervention, particularly in primary care settings.

Similarly, the prevalence of undiagnosed HTN in our cohort reflects broader epidemiological trends. Hypertension remains a major risk factor for stroke, yet a significant number of individuals with HTN are undiagnosed or inadequately controlled [12]. For instance, Mahmood et al. [13] conducted a study on 86 patients with stroke and found that 30.23% had undiagnosed HTN, which is higher than the prevalence of undiagnosed HTN in our study. Additionally, they found that patients with undiagnosed HTN had higher in-hospital mortality rates and a 30-day disability score [13]. These findings point to systemic shortcomings in routine blood pressure screening, despite well-established clinical guidelines for hypertension management.

Atrial fibrillation, although less prevalent in our study, is another important risk factor for ischemic stroke [14]. Our finding of 1.6% undiagnosed AF among stroke patients suggests that while less common, it remains a significant risk factor that requires vigilant screening, particularly in older populations.

### 4.2. Role of Primary Care and Family Doctors

Our study found that 30.9% of patients with URFs lacked an FD, emphasizing the pivotal role of primary care in stroke prevention. However, the presence of an FD was significantly associated with a higher prevalence of undiagnosed hypertension (7.6% among those with a doctor vs. 2.1% without; *p* = 0.008). These findings align with research indicating that regular primary care access is crucial for the effective management of chronic conditions [15].

The relationship between primary care access and stroke risk factor management has been well-documented. Research shows that regular primary care is associated with better outcomes in managing chronic diseases, including HTN and HLD [16]. Our findings support this, suggesting that the absence of regular primary care may contribute to the higher prevalence of URFs and subsequently increased stroke risk.

Moreover, the importance of FDs in preventive care cannot be overstated. According to the U.S. Preventive Services Task Force (USPSTF), regular screenings and management of risk factors such as HTN and HLD are essential for reducing cardiovascular events [17]. The lack of regular primary care observed in 23.97% of our study population highlights the need for improved healthcare infrastructure and policy measures to enhance access to FDs, particularly in underserved regions.

### 4.3. Geographic Disparities and Access to Care

Our study identified significant geographic disparities in the prevalence of URFs and access to primary care. Regions with higher proportions of individuals without an FD also showed a higher prevalence of URFs. This finding is consistent with research that highlights geographic variations in healthcare access and their impact on health outcomes [18].

Geographic disparities in healthcare access can lead to the unequal management of chronic conditions and, consequently, higher rates of URFs. For instance, Kassie et al. documented that rural and underserved areas often face challenges in accessing quality healthcare services, which can result in delayed diagnoses of coronary heart disease and poorer management of stroke risk factors [19]. In our study, the Du Centre-Ouest region had the highest prevalence of undiagnosed HTN (8.8%), whereas the De Est region had the highest prevalence of HLD (18.9%). Our findings suggest that addressing these geographic disparities through targeted interventions is crucial for improving stroke prevention and overall health outcomes.

Several strategies could help address these disparities, including the implementation of community-based health programs, mobile health units, and telemedicine services. Moulin et al. noted that such interventions could bridge gaps in healthcare access and facilitate early detection and management of risk factors [20]. Additionally, policies aimed at increasing the availability of primary care services in underserved areas could significantly reduce the prevalence of URFs and improve stroke prevention efforts.

### 4.4. Implications for Stroke Prevention

The high prevalence of URFs among first-ever ischemic stroke patients has important implications for stroke prevention. Public health campaigns targeting the prevention of first-ever ischemic stroke must focus on the identification and management of URFs, emphasizing the critical role of regular check-ups with FDs. The integration of routine screenings for HTN, HLD, and AF into primary care settings, as recommended by guidelines from the American Stroke Association and American Heart Association, could significantly reduce the incidence of ischemic stroke [21]. Educational initiatives should be specifically designed to highlight the importance of early detection and proactive management of these risk factors that are often undiagnosed until a stroke occurs. By improving awareness among both healthcare providers and the public about the need for routine screenings and the value of regular consultations with FDs, these campaigns can facilitate earlier identification of at-risk individuals. Enhanced access to FDs for regular check-ups ensures that risk factors are monitored and managed effectively before they contribute to a stroke. Such targeted campaigns can substantially reduce the incidence of first-ever ischemic strokes by addressing URFs and reinforcing the importance of consistent healthcare engagement. The findings highlight the importance of specific policy interventions. Policymakers should consider increasing access to FDs by creating centralized waitlist systems and providing incentives for primary care providers in underserved areas. Regional health authorities could implement targeted outreach and screening programs in regions with a high number of unattached patients, as identified in this study. Additionally, telemedicine and community-based clinics may be effective strategies to close access gaps and encourage early detection of vascular risk factors. Strengthening continuity of care and ensuring patients are attached to primary care providers should be a central focus of healthcare reform aimed at preventing strokes.

### 4.5. Limitations and Future Directions

Several limitations of our study must be acknowledged. The retrospective design and reliance on medical records may introduce biases related to incomplete data or misclassification of risk factors. Additionally, the study’s focus on a single healthcare center may limit the generalizability of the findings to other settings or populations. A major limitation is that the data were collected between 2014 and 2017, which may limit the current applicability and relevance of the findings. Although this study does not aim to provide broadly generalizable novelty, its strength lies in highlighting locally relevant insights for healthcare planning and quality improvement in Quebec, Canada. Changes in healthcare access, diagnostic practices, public awareness, and stroke prevention strategies over time may affect the prevalence and detection of risk factors today. Future research should explore prospective studies to track the development of stroke risk factors over time and assess the impact of primary care interventions on stroke outcomes. Investigations into the effectiveness of different screening strategies, as well as the role of telemedicine and other innovative approaches in improving access to care, could provide further insights into optimizing stroke prevention efforts. Additionally, studies examining the impact of specific interventions, such as community health programs and policy changes, on the prevalence of URFs could offer valuable guidance for public health initiatives.

## 5. Conclusions

Our study highlights a significant prevalence of undiagnosed risk factors (URFs) among patients experiencing their first ischemic stroke, with notable geographic disparities in access to primary care. We recommend prioritizing targeted screening programs in underserved regions, especially those with lower availability of family doctors (FDs), to facilitate the early identification and management of modifiable risk factors like hypertension and hyperlipidemia. Expanding community-based outreach and increasing the availability of FDs through policy-supported recruitment may further help reduce these disparities. Although our findings suggest a relationship between the absence of an FD and the presence of URFs, particularly hypertension, this relationship warrants confirmation through prospective studies. Additionally, the retrospective nature of our study and the use of data from 2014 to 2017 limit the generalizability of our findings to current population trends. While this study does not seek to deliver universally generalizable findings, its primary contribution lies in emphasizing insights that are particularly relevant to healthcare planning and quality improvement within the context of Quebec, Canada. Nevertheless, these results emphasize the need for a comprehensive stroke prevention strategy that includes proactive management of risk factors, equitable access to primary care, and public health interventions tailored to specific regions.

This research received no specific grant from any funding agency in the public, commercial, or not-for-profit sectors.

## Figures and Tables

**Table 1:** Distribution of gender and age.

Characteristic	Frequency (%)
Sex	
Female	257 (42.13%)
Male	353 (57.87%)
Age; (Mean ± SD)	65 ± 15

**Table 2:** Prevalence of risk factors.

Risk Factor	Frequency (n)	Percentage (%)
HTN	361/610	59.18%
DM	163/610	26.72%
HLD	354/610	58.03%
AF	92/610	15.08%
Smoking	102/610	16.72%
OSA	27/610	4.43%
CAD	91/610	14.92%
PAD	6/610	0.98%
TIA	23/610	3.77%

HTN: Hypertension; DM: Diabetes Mellitus; HLD: Hyperlipidemia; AF: Atrial Fibrillation; OSA: Obstructive Sleep Apnea; CAD: Coronary Artery Disease; PAD: Peripheral Artery Disease; TIA: Transient Ischemic Attack.

**Table 3:** Prevalence of undiagnosed risk factors.

Risk Factor	Frequency (n)	Percentage (%)
HTN	38/610	6.20%
DM	1/610	0.10%
HLD	87/610	14.30%
AF	10/610	1.60%

HTN: Hypertension; DM: Diabetes Mellitus; HLD: Hyperlipidemia; AF: Atrial Fibrillation.

**Table 4:** Distribution of Family Doctors.

Family Doctor	Frequency (%)
Yes	463 (76.03%)
No	146 (23.97%)
Total	609

**Table 5:** Distribution of Post Codes.

Post Code	Frequency (%)
De Ouest (COMTL)	183 (30%)
Du Centre-Ouest (CCSMTL)	125 (20.49%)
Du Centre Sud (CCSMTL)	90 (14.75%)
Du Nord (CMNTL)	26 (4.26%)
De Est (CEMTL)	37 (6.07%)
Outside the map	149 (24.43%)
Total	610

**Table 6:** Distribution of Post Codes with respect to family doctor.

Post Code	FD, Frequency (%)	No FD, Frequency (%)	*p*-Value
De Ouest (COMTL)	149 (81.42%)	34 (18.58%)	0.293
Du Centre-Ouest (CCSMTL)	88 (70.97%)	36 (29.03%)	
Du Centre Sud (CCSMTL)	68 (75.56%)	22 (24.44%)	
Du Nord (CMNTL)	20 (76.92%)	6 (23.08%)	
De Est (CEMTL)	25 (67.57%)	12 (32.43%)	
Outside the map	113 (75.84%)	36 (24.16%)	

FD: Family Doctor.

**Table 7:** Distribution of area codes, no family doctor and risk factors.

Area Code	Undiagnosed Risk Factor, Frequency (%)	No Risk Factor, Frequency (%)	*p*-Value
HTN			0.764
De Ouest (COMTL)	10 (5.46%)	173 (94.54%)	
Du Centre-Ouest (CCSMTL)	11 (8.80%)	114 (91.20%)	
Du Centre Sud (CCSMTL)	5 (5.56%)	85 (94.44%)	
Du Nord (CMNTL)	2 (7.69%)	24 (92.31%)	
De Est (CEMTL)	3 (8.11%)	34 (91.89%)	
Outside the map	7 (4.70%)	142 (95.30%)	
DM			0.566
De Ouest (COMTL)	0 (0%)	183 (100.00%)	
Du Centre-Ouest (CCSMTL)	1 (0.80%)	124 (99.20%)	
Du Centre Sud (CCSMTL)	0 (0%)	90 (100%)	
Du Nord (CMNTL)	0 (0%)	26 (100%)	
De Est (CEMTL)	0 (0%)	37 (100%)	
Outside the map	0 (0%)	149 (100%)	
HLD			0.629
De Ouest (COMTL)	25 (13.66%)	158 (86.34%)	
Du Centre-Ouest (CCSMTL)	21 (16.80%)	104 (83.20%)	
Du Centre Sud (CCSMTL)	15 (16.67%)	75 (83.33%)	
Du Nord (CMNTL)	3 (11.54%)	23 (88.46%)	
De Est (CEMTL)	7 (18.92%)	30 (81.08%)	
Outside the map	16 (10.74%)	133 (89.26%)	
AF			0.451
De Ouest (COMTL)	5 (2.73%)	178 (97.27%)	
Du Centre-Ouest (CCSMTL)	2 (1.60%)	123 (98.40%)	
Du Centre Sud (CCSMTL)	2 (2.22%)	88 (97.78%)	
Du Nord (CMNTL)	0 (0%)	26 (100%)	
De Est (CEMTL)	1 (2.70%)	36 (97.30%)	
Outside the map	0 (0%)	149 (100%)	

**Table 8:** Distribution of undiagnosed risk factors and no family doctor.

Undiagnosed Risk Factors	FD, Frequency (%)	No FD, Frequency (%)	*p*-Value
HTN	35 (7.56%)	3 (2.05%)	0.008 *
No HTN	428 (92.44%)	143 (97.95%)	
DM	1 (0.22%)	0 (0.00%)	1
No DM	462 (99.78%)	146 (100%)	
HLD	66 (14.25%)	20 (13.70%)	0.866
No HLD	397 (85.75%)	126 (86.30%)	
AF	7 (1.51%)	3 (2.05%)	0.709
No AF	456 (98.49%)	143 (97.95%)	

HTN: Hypertension; DM: Diabetes Mellitus; HLD: Hyperlipidemia; AF: Atrial Fibrillation; FD: Family Doctor; *: statistically significant.

## Data Availability

The original contributions presented in this study are included in the article. Further inquiries can be directed to the corresponding author.
